# Biopharmaceutical insights of particulate emulsified systems - a prospective overview

**DOI:** 10.1186/s12944-018-0757-x

**Published:** 2018-05-10

**Authors:** Jyothshna Devi Katamreddy, Prasanna Raju Yalavarthi, Subba Rao D, Sowjanya Battu, Jaya Preethi Peesa

**Affiliations:** 1 Faculty of Pharmaceutical Sciences, JNTUA, Ananthapuramu, 515002 India; 2 Department of Pharmaceutics, Krishna Teja Pharmacy College, Tirupati, 517506 India; 3 Pharmaceutics Division, Sri Padmavathi School of Pharmacy, Tirupati, 517503 India; 4 Department of Chemical Engineering, JNTUA College of Engineering, Ananthapuramu, 515002 India; 5 Department of Pharmaceutics, CMR College of Pharmacy, Hyderabad, 501401 India; 6 Department of Pharmaceutical Chemistry, Sree Vidyanikethan College of Pharmacy, Tirupati, 517102 India

**Keywords:** Lipolysis, Lymph, Mucin, OELDS, Solubilization, Transporters, Triglycerides

## Abstract

During the twenty-first century, drug discovery is expanding rapidly and a large number of chemical moieties are recognized. Many of them are poorly soluble and hence related biopharmaceutical constraints are to be addressed systematically. Among novel approaches to resolving biopharmaceutical issues, micro- and nano-emulsified systems serve as the best strategy for delivering both hydrophobic and hydrophilic drugs owing to their greater solubilization and transportation capabilities. Of late, the unique physical and biopharmaceutical properties of these liquid isotropic homogenous systems have gained substantive research importance. In addition nano/micro lipid systems share structural and functional similarity with that of the physiological lipids which offer better tolerance ability in the body. In this context, this article provides information on the historical emergence of particulate emulsified systems, importance and rationale of selection of carriers. It also encompasses the physicochemical principles that are responsible for the elevation of therapeutic outcomes of delivery systems. Detailed and schematic absorption of these drug delivery systems is explained here. Gastro-intestinal biochemistry necessary in the understanding of digestion process, lipolytic products formed, micellar structures, enzymes, transporters, mechanism of cell uptake involved after subsequent oral absorption are also emphasized. In addition, this article also explains disposition and pharmacokinetic properties of emulsified systems with real-time therapeutic research outcomes. The influence of biochemical compositions and biopharmaceutical principles on absorption and disposition patterns of ME/NEs was described in the article for the interest of readers and young researchers.

## Background

With the advent of several emerging novel chemical entities, delivery of high molecular weight and poorly soluble bioactive molecules remains a challenging task. Therapeutic efficacy of low soluble molecules (BCS Class II and IV) is generally high and hence the design of oral delivery systems with improved dissolution and permeability characteristics cannot be ignored [1] However, poor solubility characters pose greater inter/intra subject variabilities and dose disproportionalities. Yet, most of the novel strategies still remain open for developing versatile oral drug delivery systems since the route of administration is salient [2, 3]. However, development of stable and acceptable dosage forms for lipophilic drugs is an ever challenging task for the researchers.

In times of yore, many attempts were made to deliver lipophilic/hydrophobic moieties for better therapeutic outcomes. Of late, Oral Emulsified Lipid Delivery Systems (OELDS) was instigated with sulphonamide lipid emulsions [4]. In such systems, incorporation of poorly water-soluble drugs into the inert core of carriers composed of oils, surfactants, and cosolvents, enables them to solubilize in GI fluids by forming a colloidal dispersion. Microemulsions, nanoemulsions, Self Microemulsifying/Nano emulsifying Drug Delivery Systems (SMEDDS/SNEDDS), Solid lipid nanoparticles (SLN’s), Nanoparticulate lipid structures (NLS) are recognized as frontline approaches in OELDS [5, 6]. Amongst these, particulate micro/nanoemulsified systems are particularly germane to communication are fundamental approaches for revolutionary strategies have gained good research due to their potentials in increasing both lymphatic and portal circulation [7]. Nevertheless, therapeutic outcomes of OELDS are affected by particle/droplet size, the rate of dispersion, emulsification time and precipitation of drug in GIT. Moreover, dietary edible oils which exhibit typical food effect on absorption of drugs owing to structural and functional similarities with that of the physiological lipids, offer better tolerance.

Based on the potential considerations of ME/NEs as an appealing substitute, the review was undertaken to emphasize typical biopharmaceutical aspects of oral absorption, including enzymes, transporters, and carriers involved therein. Distribution and elimination attributes of ME/NEs are also discussed here. Although both the emulsified particulate systems differ in droplet size, composition, and physicochemical properties, there is a firm connection in their in-vivo performance. Therefore, both the emulsion systems and their subtypes, self-micro/nano emulsifying drug delivery systems are explicated together.

### Micro/Nanoemulsified systems

Findings of Hoar and Schulman in 1940’s have led to the concept of the microemulsion, which formed a clear homogenous fluid of milky emulsion with hexanol. Later, the term microemulsion was coined by Schulman in 1959 based on the droplet size (100–600 nm). Since then the term has modified and defined, according to, on many occasions [8].

Micro/nanoemulsions (ME/NEs) share the similarities in definition, as they are isotropic, homogenous liquid systems composed of oil, amphiphile and water contents, very often included with a modifier like cosurfactant or cosolvent [9] to constitute stability. Unfortunately, these emulsified lipid systems are used as misnomers on several occasions but they differ majorly in terms of droplet size. Nanoemulsions as dispersed oil droplets with 10–100 nm radii are thermodynamically non-equilibrium and kinetically stable systems [10]. Microemulsion composed of high amphiphile concentration (> 20%) offers thermodynamic stability. The relative size of ME/NEs droplets accounts for their optical transparency, translucence, and stability [11]. Owing to sub-micron droplet size, the solubility of lipophilic drugs is enhanced and fortified with intra-luminal processing [12]. The release of encapsulated functional components from ME/NEs is potentiated by trigger mechanisms such as pH, ionic strength, temperature and presence of enzymes. It is characterized in terms of increase in concentration in the continuous phase or target location such as the mouth, nose, stomach, or gastrointestinal tract with the function of time [11].

ME/NEs including SMEDDS and SNEDDS are strategically significant due to the protection of lipophilic drugs against degradation in GIT, translocation across epithelial barriers by extending transit time and specified absorptive pathways. Performance of ME/NEs is typically depended on the class/type of system and composition [13].

### Components and classification of ME/NEs

#### Oils

Oils as carriers firstly meant for hydrophobic drugs to sink, long, medium and short chain triglycerides (LCT, MCT, and SCT) were predominantly used in the preparation of ME/NEs with the rank order of MCTs>LCTs>SCTs [1]. Type of oil used in the formulation has a great impact on solubilization, digestion of carrier lipid and drug release with desired characteristics. Principally, LCTs and MCTs are converted into free fatty acids by water dispersion during digestion. Free fatty acids of MCTs are able to migrate rapidly into surrounding medium whereas those produced by LCTs accumulate at the oil-water interface thereby lipase activity is inhibited [1, 11] Semisynthetic MCT’s are superior over natural MCTs as it was proven in Naringenin nanoemulsions [3, 14]. Combination of LCT’s and MCTs was also promised with superior emulsifying and dissolution profiles [15]. The success of MEs/NEs is affected by molecular and lipid characters of MCT and LCTs such as loading of drug and droplet size, chemical nature, and stability [16, 17]. As absorption of ME/NEs is governed by Ostwald ripening principle, significant portal absorption of crodomol GTCC, an MCT and lymphatic absorption of corn and canola oils (LCT) were resulted [1, 18].

#### Amphiphiles

Due to typical structural significance, amphiphiles reduce the interfacial tension and impart thermodynamic stability in dispersions. Droplet size of hetero-dispersions is affected by HLB value, type, and concentration of surfactant [3]. Surfactants with high HLB (12–18) are suitable candidates for ME/NE’s [19], since their ability to form smaller droplets within the GI lumen that enables the absorption maxima. In contrast, at higher concentrations, surfactants hamper the absorption process due to increased apparent weight on whole [1].

Being nonionic, polysorbate is less toxic and unaffected by changes in pH, ionic strength and promotes the formation of ultrafine droplets [20] whereas, poloxamers display aggregation with Ostwald ripening. But in combination with ionic surfactants, due aggregation of poloxamers is minimized by imparting a positive charge on the surface. A similar principle was noticed in a binary mixture of DDAB and C12E5 DDAB (cationic –nonionic) surfactants which had no obvious effect on droplet structure and size, although it improved the stability of nanoemulsion [21]. Hence, the nonionic surfactants are considered to be stable in the design of ME/NEs and their subtypes [3]. Tween 80, cremophor EL and cremophor RH40 had proven their superiority on digestion and increased absorption by inhibiting the efflux transporters such as p-gp, breast cancer resistance protein and MDR-protein [4, 22, 23]. A recent success of amphiphiles was portrayed with a combination of Gelucire®, sucrose ester, a non-digestable surfactant. The substitution of the non-digestible surfactants with digestible surfactants, sucrose esters S-1670 and Span® 60, eliminated the digestion lag time with no change in the formation of colloidal structures [24].

#### Cosurfactants /Cosolvents

Short-to-medium-chain (C3-C8) alcohols such as methanol, ethanol, isopropyl alcohol, carbitol, PEG’s, glycerin, propylene glycol, etc. have the potential to increase the emulsification efficiency by reducing the bending stress at an interface and allow film flexibility to different curvatures for the formation of ME/NE’s. They primarily increase the mobility of hydrocarbon tail and allow greater penetration of oil at the interface [25, 26]. Transcutol, a cosurfactant has efficiently increased the solubility of paracetamol as a function of temperature over other cosolvents and also combinations [27].

### Biochemistry of gastrointestinal tract

Mucins, long (200–1000 nm) heterogenous polydispersed filaments of glycosylated proteins are protecting GI mucosa from pH fluctuations, pathogens, mechanical irritation and toxins [28, 29]. They are categorized into membrane-bound and secretory mucins [30]. Majority of mucins are linked with proline, rich in serine and /or threonine glycosylation sites repeated in tandem (named as tandem repeats). These repeated units are widely varied between mucin types in terms of length (5–375 amino acids) and in number (5–395 repeats) [31]. Each epithelial mucin gene discovered was named as MUC in 1990. Currently, in humans, 20 mucin-type glycoproteins have been assigned to the MUC gene family, as approved by the Human Genome Organization- Gene Nomenclature Committee (HUGO/GNC). MUC1, MUC3A, MUC3B, MUC4, MUC12, MUC13, MUC15, MUC16, MUC17, MUC18, and MUC20 mucins have trans-membrane domains in their C-terminus and thus are type 1 membrane proteins [32]. Stomach epithelia contain MUC1, which is present on the apical membranes of parietal cells in fundic glands [33]. MUC2 contains four conserved cysteine-rich domains that have sequence similarity to the D-domains of pro-von Willebrand clotting factor expressed in goblet cells of the intestine [34, 35]. The other large gel-forming cysteine-rich secretory mucins are MUC5AC, MUC5B, MUC6, and MUC19 [36]. The MUC 3 gene was essentially expressed in the epithelial cells of small intestine especially in goblet cells and in enterocytes [37]. Majorly, MUC 5 AC and MUC 6 gene are expressed in gastric tracts. Mucins affect the absorption of ME/NEs in terms of their biochemical composition, viscosity, diffusivity, dipole moment, zwitterion profiles, ionic strength and interaction with the dispersion medium. Overall, ME/NEs undergo absorption at various sites of GIT such as gastric lumen, stomach, and small intestine as their biomembrane diffusion is proportional to the reciprocal of mucin concentration [36].

### In-vivo drug solubilization process

The solubility of lipophilic drugs at absorption site depends on selected lipid vehicles as they enhance solvation capacity of the GI fluids by forming swollen colloidal micelles. These solubilized forms of drugs (intact micelles) undergo certain mesomorphic transformations which are due to during dispersion of GIT contents, dilution of contents by various endogenous secretions and effect of drug-chemical stability by various digestive processes. Absorption of ME/NEs takes place from the free drug concentration that is in equilibrium with the solubilized colloidal reservoir rather than from intact micelles [4].

#### Absorption of ME/NEs

**Fig. 1:**
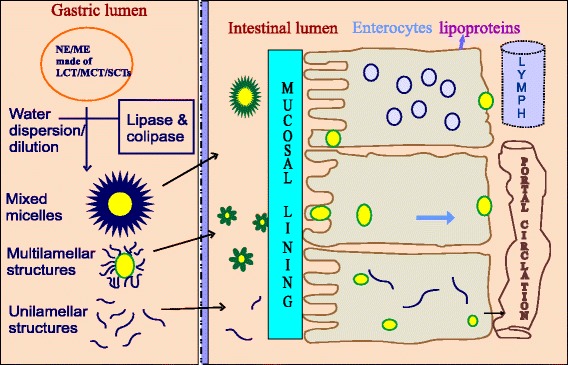
Steps in intestinal absorption of ME/NEs

Mostly, absorption of ME/NEs is high in small intestinal lumen rather than in other parts of GIT since it contains biliary lipids (bile salts, phospholipids, and cholesterol). MCTs with amphiphilic nature present in particulate emulsified systems undergo lipolysis at lumen of the intestine into free fatty acids and monoglycerides. These products have the ability to coalesce readily with bile lipids and form micelles. Further, fatty acids of 10–12 carbon atoms and drugs with lop *P* < 5 passively diffused from the lumen into the systemic circulation by enterocytes [38–41]. Fatty acids of more than 10–12 carbon atoms and drugs with log *P* > 5 are re-esterified to triglycerides in enterocytes as represented in Fig. 1. Cholesterol also undergoes esterification by luminal enzymes into cholesterol esters. The formed triglycerides and cholesterol esters are then covered by a layer of proteins and phospholipids to form chylomicrons, which leaves the cell through exocytosis and enter the lymphatics owing to apparent molar volume increase. Therefore, drugs undergo either portal or lymphatic absorption depending on molecular features and diffusion coefficient of API [42–47].

A pH-stat automatic titration unit model demonstrated the lipolysis of ME/NEs and subsequent phases which alters the release profile and fate of a drug in GIT. The further extent of digestion affects release profiles of the drug located in the core of ME/NEs rather than shell [18, 48]. Carriers of ME/NEs transport the drug present in oil core to mucus layer while the drug available in surfactant shell get separate out as free molecules from carriers and diffuse directly to the epithelium. Though lipophilic compounds readily diffuse across the apical plasma membrane of intestinal epithelium the subsequent passage across the basolateral membrane and into blood are by no means guaranteed [49]. Thus, ME/NEs facilitate improved transport across the intestinal wall and lead to lowering the effect of protease, an intestinal enzyme and sustained plasma uptake [50, 51], this attribute was proven to be successful with the sustained release of rhPTH1–34, a peptide drug by opening tight junctions [52, 53].

ATP-binding cassette (ABC) transporters and solute carriers are notable intestinal transporters which regulate the transport of molecules into and out of enterocytes. Cytochrome P-450 3A4 (CYP3A4), multidrug efflux pump and P-glycoprotein (P-gp) are present high levels at villus tip enterocytes of the small intestine. P-gp is ABC transporter and highly expressed on the brush-border membrane of columnar epithelial cells. The efflux transport of particulate emulsified systems into enterocytes is affected by these local transporters. However, efflux inhibition is anticipated with membrane fluidization, induced conformational changes and cholesterol depletion [54]. PEG 400, Cremophor EL, RH40 and combinatorial surfactants are successful in inhibiting P-gp by forming the micelles during emulsification which can masquerade drug thereby not exposing core drug molecules to the efflux transporters [55–60].

Cellular surfaces are dominated by negatively charged sulfated proteoglycans that play an important role in cellular proliferation, migration, and motility. Particulate emulsified systems with higher surface charge, bind strongly to the cell membrane with electrostatic interactions between the anionic membrane and cationic nanodroplets, thus increasing the cellular uptake. After adsorption onto the cellular membrane, the uptake occurs possibly via pinocytosis, nonspecific or receptor-mediated endocytosis or phagocytosis [61–63]. Absorption of particulate emulsified systems is affected by surface charges (either positive or negative) present on ME/NEs and this phenomenon was evidently demonstrated by griseofulvin emulsions. Therefore, the zeta potential of ME/NEs certainly affects the pharmacokinetics of isotropic systems [64].

#### Lymphatic transport of ME/NEs

M cells of the lymphoid follicle-associated epithelium represent a potential portal for intestinal drug delivery because of their high transcytotic capacity and ability to transport particulates [65]. Particle uptake involves contact with microvilli on the M cell surfaces, which is followed by rapid phagocytosis through an extension of apical membrane. The phagocytic vesicle fuses with basolateral membrane before transferring its contents to the intraepithelial compartment. Alternately, the particles are engulfed by phagocytic cells into compartments or pass through basal lamina to the subepithelial region and thus the particulate emulsified systems drained into lymph [48].

#### Lymphatic uptake

**Fig. 2:**
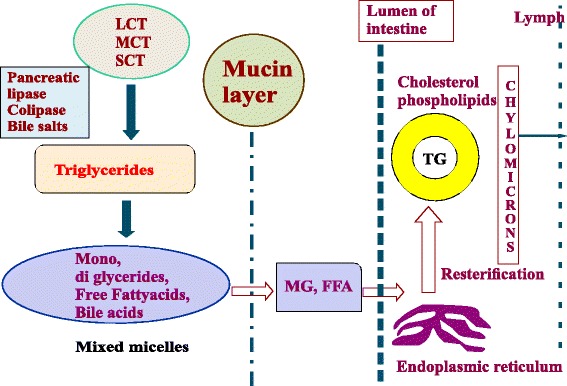
Lymphatic uptake of ME/NEs

As demonstrated in Fig. 2, a fraction of particulate drug, encapsulated by triglycerides is restructured in the endoplasmic reticulum and then released into the mesenteric lymph duct as chylomicrons [66]. Thereafter it reaches the systemic circulation via thoracic lymph [67–69]. Lymphatic uptake becomes ever effective since the drug molecules are bypassed. But, the chain length of triglycerides and solubility of the drug in oil determines the outcome of lymphatic uptake of orally administered lipophilic moieties.

#### Distribution of ME/NEs

Being submicron sized systems, ME/NEs exhibit prolonged circulation [70]. A major portion of orally administered ME/NEs undergo intestinal lymphatic absorption followed by biodistribution and finally ended up with lymphatic drainage to capillaries. An extent of the biodistributed drug is affected by the macrophages of liver, spleen, and lungs with the result of RES uptake [71]. HCO-60, a pharmaceutically modified castor oil in conjugated linoleic acid microemulsion was effective as co-surfactant against RES uptake [72]. Combination of carriers such as capmul and myvacet oil used thereof in particulate systems accounts largely for biodistribution of emulsified systems [73, 74]. A potential intestinal P-gp inhibitor tween 80 was also reported to produce higher indinavir distribution levels in plasma. However, highly hydrophilic drug undergo rapid uptake by organs of the reticulo-endothelial system (RES) lead to lowering of drug levels [75].

#### Elimination of ME/NEs

Emulsions poor in cholesteryl ester but rich in free cholesterol show remnant-like behavior, whereas emulsions with rich cholesteryl ester but poor in free cholesterol were metabolized like nascent chylomicron particles. Emulsions of defined composition and with known metabolic behavior should be of value not only to probe lipoprotein metabolism but perhaps also as vehicles for the delivery of hydrophobic drugs to targeted organs. Subsequent dosing of emulsion, droplets acquire apoproteins (apo-E, apo-CI, apo-CII, apo-CIII, and apo-AIV) from high-density lipoproteins and very low-density lipoproteins of blood. Apo-CII acts as lipoprotein lipase (LPL) activator, which leads to hydrolysis of triglycerides, whereas apo-E helps in hepatic removal of remnants [76].

ME/NEs, being prolonged circulation entities display regular elimination patterns with increased half-life generally. Droplet sizes of 50–100 nm were removed by the liver and spleen, polymeric micelles smaller than 5 and 5–10 nm were easily eliminated through the renal glomeruli [77]. Such kind of variability in elimination process is attributed due to diminished bile flow, poor gastric emptying, and disturbances in intestinal and hepatic functions [78].

#### Molecular actions and outcomes of ME/NEs

Particulate emulsified delivery systems are being gradually more used to protect bioactives against the intense environment, to improve molecular targets, stability and to increase bioavailability. Molecular action of selected lipophilic drugs when associated with oil core or amphiphiles of the ME/NE systems is ascribed to transfer the drug molecules into the targeted cells by fusion of oil droplet with the cell membranes through lipid exchange or by endocytosis of the oil droplets [79]. If the targeting moieties (folate, thiamine, etc.) with corresponding cell surface receptors are anchored to ME/NEs to adapt multifunctional property, they can directly target a tissue of interest and remain longer at the disease site to allow the total transfer of the drug molecules. Different molecules owned by anti-inflammatory agents, insulin, cancer etc., were rationalized herein.

Essential PUFA transporters of an abluminal membrane of the endothelial cells of BBB allowed the CNS acting essential PUFA (omega-3 and omega-6), a lipophilic moiety into the brain [80]. Nonionic surfactants (tween 80 and poloxamer) augmented brain targeting essential PUFA through lipoprotein receptor-mediated uptake owing to the affinity of apolipoprotein E for LDL receptor in the BBB enabled uptake by RME (receptor-mediated endocytosis). In another instance, enhanced brain uptake of doxorubicin resulted with drug- loaded MEs with principal interaction of ME entities with plasma apolipoprotein A–I facilitated scavenger receptor in BBB endothelium [81].

Curcumin encapsulated by a combination of cationic surfactants such as cetyl and dodecyl trimethylammonium bromide was found to be intact even against alkaline hydrolysis [82] and bioaccessible with use of triacylglycerols (medium chain) [83]. Polymeric micelles of curcumin were bestowed with over 55-fold increase in bioavailability [84]. In similar lines, tamoxifen loaded NEs (size < 47 nm) inhibited cell proliferation 20-fold greater and increased cell apoptosis 4-fold greater in the HTB-20 breast cancer cell line [85].

Oral administration of ME of cyclosporine A resulted in increased bioavailability with not many side effects in hepatic impaired patients [86] and stable plasma drug concentrations were maintained in renal impaired patients [87]. Oral application of carvedilol loaded NEs has displayed two-fold faster release profile and several folds increase in C_max_ and AUC [88]. Thus the particulate emulsified systems are proven to be superior in the treatment of certain ailments though they were orally administered.

## Conclusion

ME/NEs are the most dynamic and novel appealing platforms for poorly soluble moieties with expected therapeutic outcomes. In view of the fact, development of ME/NEs is not an easy task for the formulators wherein, they require utmost attention on selection and usage of carriers/integrants of the delivery system. Thus, this article prolifically presents the information on carriers, composition and their in-vitro and in-vivo functions. It was successful to illustrate on the micellization of particulates with luminal contents and subsequent intestinal/lymph absorption. Impact of biochemical and biopharmaceutical principles on absorption and disposition patterns of ME/NEs was presented well herein for the interest of readers and young researchers.

## Abbreviations

API: Active pharmaceutical ingredient
DDAB: Didodecyl dimethyl ammonium bromide
GIT: Gastrointestinal tract
HLB: Hydrophilic lipophilic balance
HUGO/GNC: Human genome organization- gene nomenclature committee
LCT: Long chain triglycerides
MCT: Medium chain triglycerides
MDR: Multi drug resistance
ME/NEs: Microemulsions/nanoemulsions
MUC: Mucin
NLS: Nanoparticulate lipid structures
OELDS: Oral emulsified lipid delivery systems
PEG: Polyethylene glycol
RES: Reticulo-endothelial system
RMS: Receptor-mediated systems
SCT: Short chain triglycerides
SLN’s: Solid lipid nanoparticles
SMEDDS: Self microemulsifying drug delivery systems
SNEDDS: Self nanoemulsifying drug delivery systems
